# Identification of Birds through DNA Barcodes

**DOI:** 10.1371/journal.pbio.0020312

**Published:** 2004-09-28

**Authors:** Paul D. N Hebert, Mark Y Stoeckle, Tyler S Zemlak, Charles M Francis

**Affiliations:** **1**Department of Zoology, University of GuelphGuelph, OntarioCanada; **2**Program for the Human Environment, Rockefeller UniversityNew York, New YorkUnited States of America; **3**National Wildlife Research Centre, Canadian Wildlife ServiceOttawa, OntarioCanada

## Abstract

Short DNA sequences from a standardized region of the genome provide a DNA barcode for identifying species. Compiling a public library of DNA barcodes linked to named specimens could provide a new master key for identifying species, one whose power will rise with increased taxon coverage and with faster, cheaper sequencing. Recent work suggests that sequence diversity in a 648-bp region of the mitochondrial gene, cytochrome *c* oxidase I (COI), might serve as a DNA barcode for the identification of animal species. This study tested the effectiveness of a COI barcode in discriminating bird species, one of the largest and best-studied vertebrate groups. We determined COI barcodes for 260 species of North American birds and found that distinguishing species was generally straightforward. All species had a different COI barcode(s), and the differences between closely related species were, on average, 18 times higher than the differences within species. Our results identified four probable new species of North American birds, suggesting that a global survey will lead to the recognition of many additional bird species. The finding of large COI sequence differences between, as compared to small differences within, species confirms the effectiveness of COI barcodes for the identification of bird species. This result plus those from other groups of animals imply that a standard screening threshold of sequence difference (10× average intraspecific difference) could speed the discovery of new animal species. The growing evidence for the effectiveness of DNA barcodes as a basis for species identification supports an international exercise that has recently begun to assemble a comprehensive library of COI sequences linked to named specimens.

## Abstract 2

Can a single gene be used to identify all different animal species? An analysis of 255 North American birds shows that cytochrome *c* oxidase I might have this potential

## Introduction

The use of nucleotide sequence differences in a single gene to investigate evolutionary relationships was first widely applied by Carl Woese (Woese and Fox 1977). He recognized that sequence differences in a conserved gene, ribosomal RNA, could be used to infer phylogenetic relationships. Sequence comparisons of rRNA from many different organisms led initially to recognition of the *Archaea,* and subsequently to a redrawing of the tree of life. More recently, the polymerase chain reaction has allowed sequence diversity in any gene to be examined. Genes that evolve slowly, like rRNA, often do not differ among closely related organisms, but they are indispensable in recovering ancient relationships, providing insights as far back as the origin of cellular life (Woese 2000). On the other hand, genes that evolve rapidly may overwrite the traces of ancient affinities, but regularly reveal divergences between closely related species.

Mitochondrial DNA (mtDNA) has been widely employed in phylogenetic studies of animals because it evolves much more rapidly than nuclear DNA, resulting in the accumulation of differences between closely related species (Brown et al. 1979; Moore 1995; Mindell et al. 1997). In fact, the rapid pace of sequence change in mtDNA results in differences between populations that have only been separated for brief periods of time. John Avise was the first to recognize that sequence divergences in mtDNA provide a record of evolutionary history within species, thereby linking population genetics and systematics and establishing the field of phylogeography (Avise et al. 1987). Avise and others also found that sister species usually show pronounced mtDNA divergences, and more generally that “biotic entities registered in mtDNA genealogies…and traditional taxonomic assignments tend to converge” (Avise and Walker 1999). Although many species show phylogeographic subdivisions, these usually coalesce into single lineages “at distances much shorter than the internodal branch lengths of the species tree” (Moore 1995). In other words, sequence divergences are much larger among species than within species, and thus mtDNA genealogies generally capture the biological discontinuities recognized by taxonomists as species. Taking advantage of this fact, taxonomic revisions at the species level now regularly include analysis of mtDNA divergences. For example, many newly recognized species of birds have been defined, in part, on the basis of divergences in their mtDNA (e.g., Avise and Zink 1988; Gill and Slikas 1992; Murray et al. 1994; AOU 1998; Banks et al. 2000, 2002, 2003).

The general concordance of mtDNA trees with species trees implies that, rather than analyzing DNA from morphologically identified specimens, it could be used the other way around, namely to identify specimens by analyzing their DNA. Past applications of DNA-based species identification range from reconstructing food webs by identifying fragments in stomachs (Symondson 2002) to recognizing products prepared from protected species (Palumbi and Cipriano 1998) and resolving complexes of mosquitoes that transmit malaria and dengue fever (Phuc et al. 2003). Despite such demonstrations, the lack of a lingua franca has limited the use of DNA as a general tool for species identifications.

If a short region of mtDNA that consistently differentiated species could be found and accepted as a standard, a library of sequences linked to vouchered specimens would make this sequence an identifier for species, a “DNA barcode” (Hebert et al. 2003a). Recent work suggests that a 648-bp region of the mitochondrial gene, cytochrome *c* oxidase I (COI), might serve as a DNA barcode for the identification of animal species. This gene region is easily recovered and it provides good resolution, as evidenced by the fact that deep sequence divergences were the rule between 13,000 closely related pairs of animal species (Hebert et al. 2003b). The present study extends these earlier investigations by testing the correspondence between species boundaries signaled by COI barcodes and those established by prior taxonomic work. Such tests require the analysis of groups that have been studied intensively enough to create a firm system of binomials; birds satisfy this requirement. Although GenBank holds many bird sequences, these derive from varied gene regions while a test of species identification requires comparisons of sequences from a standard gene region across species. Accordingly, the barcode region of COI was sequenced in 260 of the 667 bird species that breed in North America (AOU 1998).

## Results

**Figure 1:**
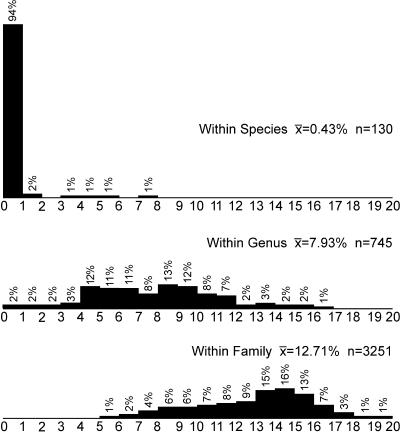
Comparison of Nucleotide Sequence Differences in COI among 260 Species of North American Birds Pairwise comparisons between 437 COI sequences are separated into three categories: differences between individuals in the same species, differences between individuals in the same genus (not including intraspecific differences), and differences between individuals in the same family (not including intraspecific or intrageneric differences).

All 260 bird species had a different COI sequence(s); none was shared between species. COI sequences in the 130 species represented by two or more individuals were either identical or most similar to other sequences of the same species. Furthermore, with a few interesting exceptions discussed below, COI sequence differences between closely related species were far higher than differences within species (18-fold higher; average Kimura-2-parameter [K2P] differences between and within species, 7.93% and 0.43%, respectively) (Figure 1).

**Figure 2:**
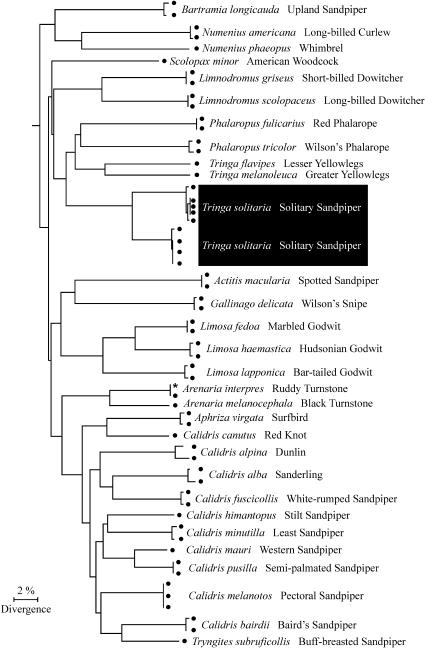
NJ Tree of COI Sequences from 30 Species in Family *Scolapacidae* (Sandpipers and Kin) The divergent pair of clustered sequences of Tringa solitaria is highlighted. An asterisk indicates a COI sequence from GenBank.

**Figure 3:**
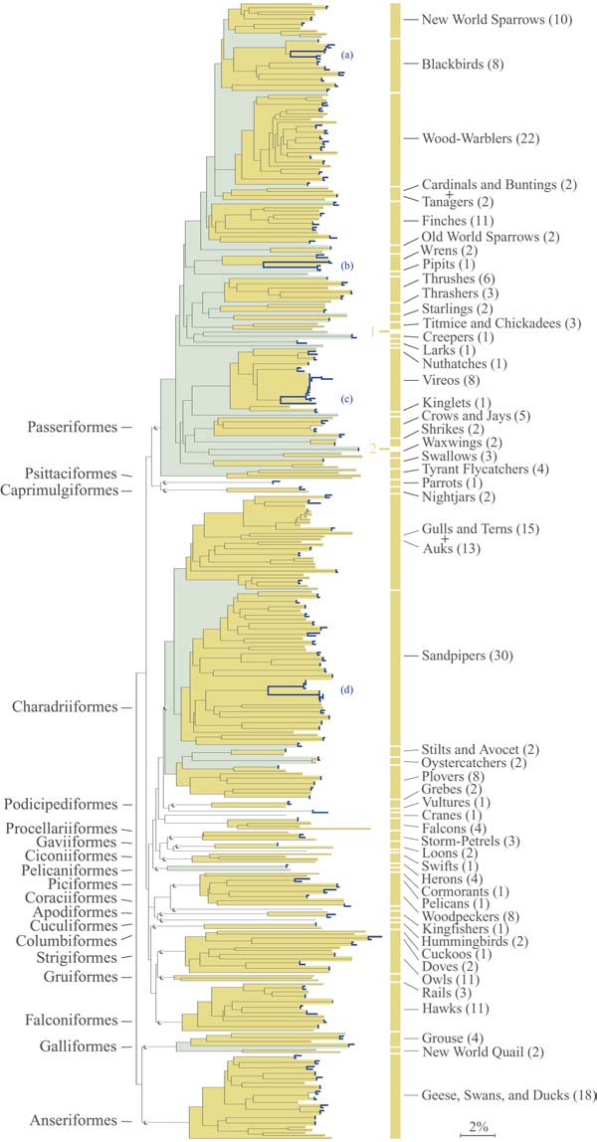
NJ Tree of COI Sequences from 260 Species of North American Birds Intraspecific divergences were sampled in 130 species; these are marked in blue. Four species showed deep intraspecific divergence: (a) *Sturnella magna,* (b) *Cistothorus palustris,* (c) *Vireo gilvus,* and (d) *Tringa solitaria.* Higher-order classifications in families (gray) and orders (gold) are highlighted, and are labeled on the left and right of the figure, respectively. Gold numerals indicate the two species that appear as paraphyletic lineages at the family level: (1) Oenanthe oenanthe and (2) *Hirundo rustica.*

In most cases the neighbor-joining (NJ) tree showed shallow intraspecific and deep interspecific divergences (Figure 2). However, in four exceptional cases, there were deep divergences within a species (*Tringa solitaria,* Solitary Sandpiper; *Sturnella magna,* Eastern Meadowlark; *Cisthorus palustris,* Marsh Wren; and *Vireo gilvus,* Warbling Vireo). COI sequences in each of these polytypic species separated into pairs of divergent clusters in the NJ tree. The intraspecific K2P distances in these exceptional species were 3.7%–7.2%, 9- to 17-fold higher than the average distance (Figures 2, 3, and S1).

Setting aside these polytypic species, the average intraspecific distance was very low, 0.27%, and the maximum average intraspecific difference was only 1.24%. Most congeneric species pairs showed divergences well above this value, but 13 species in four genera had interspecific distances that were below 1.25%. They included *Larus argentatus, L. canus, L. delawarensis, L. glaucoides, L. hyperboreus, L. marinus,* and L. thayeri (Herring Gull, Mew Gull, Ring-billed Gull, Iceland Gull, Glaucous Gull, Great Black-Backed Gull, and Thayer's Gull); Haematopus bachmani and H. palliatus (Black Oystercatcher and American Oystercatcher); Corvus brachyrhynchos and C. caurinus (American Crow and Northwestern Crow); and Anas platyrhynchos and A. rubripes (Mallard and American Black Duck) (Figure S1).

Although species were the focus of this study, we noted that the NJ tree of COI sequences generally matched avian classifications at higher levels, with most genera, families, and orders appearing as nested monophyletic lineages concordant with current taxonomy (Figures 3 and S1).

## Discussion

The simplest test of species identification by DNA barcode is whether any sequences are found in two species; none was in this study. Although sequences were not shared by species, sequence variation did occur in some species. Thus the second test is whether the differences within species are much less than those among species. In this study we found that COI differences among most of the 260 North American bird species far exceeded those within species.

**Figure 4:**
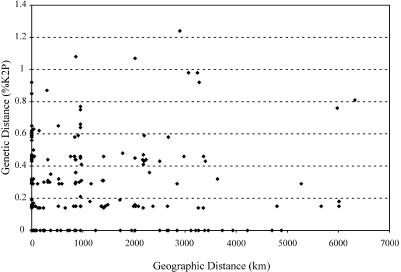
Genetic Difference versus Geographic Distance For each same-species pair of specimens, the geographic distance between where specimens were collected is plotted against their COI divergence (K2P).

In order to conservatively test the effectiveness of COI barcodes as an identification tool, our sample must not have underestimated variability within species or have overestimated it among species. Our measures of intraspecific variation could be underestimates if members of a species show sequence divergence across their distribution that our study failed to adequately register. The two to three representatives of the 130 species used to examine this issue were collected from sites that were, on average, approximately 1,080 km apart, suggesting adequate representation of genetic diversity across their ranges. However, to further investigate this issue, we compared sequence differences within species to geographic distances between the collection points for their specimens and found these were unrelated (Figure 4). Based on these results, high levels of intraspecific divergence in COI in North American birds appear uncommon, given that we analyzed 130 different species in a variety of orders. Our findings are supported by a review of 34 mostly North American birds which showed a similarly low average maximum intraspecific K2P divergence of mtDNA of 0.7% (Moore 1995). Similarly, Weibel and Moore (2002) reported an average intraspecific divergence of 0.24% in their study of COI variation in woodpeckers. We conclude that our investigation has not underestimated intraspecific variation in any systematic fashion.

On the other hand, our discovery of four polytypic species within a sample of 130 makes it likely there are other North American birds with divergent populations that may represent hidden species. Recent studies have identified marked mtDNA divergences within North American populations of Common Ravens (Omland et al. 2000), Fox Sparrows (Zink and Weckstein 2003), and Curve-billed Thrashers (Zink and Blackwell-Rago 2000), leading to proposals to split each into two or more species. Species with Holarctic distributions are particularly good candidates for unrecognized species, and recent DNA and morphological investigations have led taxonomists to split several such species into two, including Wilson's and Common Snipes, American and Eurasian Three-toed Woodpeckers, and American and Water Pipits (Zink et al. 1995, 2002; Miller 1996; AOU 1998; Banks et al. 2000, 2002, 2003). Widespread application of COI barcodes across the global ranges of birds will undoubtedly lead to the recognition of further hidden species.

**Figure 5:**
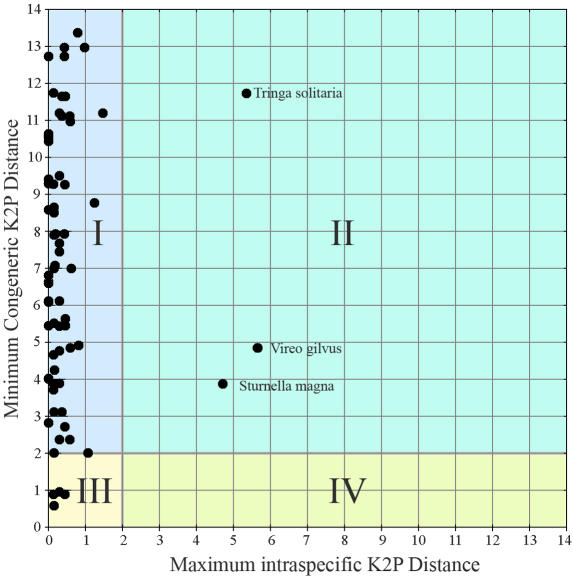
Intraspecific Compared to Interspecific COI Distances (K2P) for Individual Species For each species in which this comparison was possible (*n* = 73), maximum intraspecific variation is compared to minimum interspecific congeneric difference. For illustration purposes shown here, 2.0% is chosen as a cutoff between usual values for intra- and interspecific variation. This divides the graph into four quadrants that represent different categories of species: (I) Intraspecific distance, <2%; interspecific distance, >2%: concordant with current taxonomy; (II) Intraspecific distance, >2%; interspecific distance, >2%: probable composite species (i.e., candidate for taxonomic split); (III) Intraspecific distance, <2%; interspecific distance, <2%: recent divergence, hybridization, or synonymy; (IV) Intraspecific distance, >2%; interspecific distance, <2%: probable misidentification of specimen.

Any critical test of the effectiveness of barcodes must also consider the possibility that our study has overestimated variability among species. We therefore looked at species individually, comparing their minimum distance to a congener with the maximum divergence within each species. This analysis included a number of well-recognized sibling species, including Calidris mauri and *C. pusilla, Fraternicula arctica* and *F. corniculata,* and *Empidonax traillii* and *E. virescens.* There were sufficient data to perform this analysis on three of the four polytypic species and on 70 of the 126 remaining species (Figure 5). The average maximum K2P divergence within these 70 species was 0.29%, while the average minimum distance to a congener was 7.05% (24-fold higher), values comparable to those for the entire data set. Prior studies that looked exclusively at sister species of birds found an average K2P mtDNA distance of 5.1% in 35 pairs (Klicka and Zink 1997) and 3.5% in 47 pairs (Johns and Avise 1998). More generally, 98% of sister species pairs of vertebrates were observed to have K2P mtDNA divergences greater than 2% (Johns and Avise 1998). Thus it appears that a COI barcode will enable the separation of most sister species of birds.

There is a possibility that the North American bird fauna is not representative of the global situation. The recent and extensive glaciations in North America may have decreased within-species variability by inducing bottlenecks in population size or may have increased variation between species by pruning many sister taxa (Avise and Walker 1998; Mila et al. 2000). This issue can only be resolved by evaluating the efficacy of barcodes in tropical and southern temperate faunas to ascertain if our results are general. We note that recent mtDNA studies in these settings have found both multiple sibling species in what were thought to be single species (Ryan and Bloomer 1999) and geographically structured variation suggesting the presence of cryptic species (Hackett and Rosenberg 1990; Bates et al. 1999).

The diagnosis of species is particularly difficult when they are young. Moreover, hybridization is often common when the ranges of recently arisen species overlap, further complicating identifications. Such newly emerged species are sometimes referred to as superspecies (Mayr and Short 1970), or species complexes, to indicate their close genetic similarity. For example, the white-headed gulls are thought to have diverged very recently, some less than 10,000 years ago (Crochet et al. 2002, 2003), and hybridization is common among many of them. It is thus not surprising that their COI barcodes and other gene loci are very similar. DNA barcodes can help to define the limits of such recently emerged species, but more gene loci need to be surveyed and more work is required to determine which analytical methods can best deduce species boundaries in such cases. The NJ method used here has the advantage of speed, and performs strongly when sequence divergences are low, so it is generally appropriate for recovering intra- and interspecies phylogeny. However, a library of COI barcodes linked to named specimens will provide the large data sets needed to test the efficacy of varied tree-building methods (for review, see Holder and Lewis 2003).

Even between species that diverged long ago, hybridization will lead to shared or very similar sequences at COI and other gene loci. Because mitochondrial DNA is maternally inherited, a COI barcode will assign F_1_ hybrids to the species of their female parent. Hybridization leading to the transfer of mtDNA from one species to another can result in a mtDNA tree that is incongruent with the species tree, but it will not necessarily prevent species from being distinguished, unless the mitochondrial transfer is so recent that their sequences have not diverged (Moore 1995). However, recent hybridization will lead species to share COI barcodes, and we expect that more intensive study will reveal such shared sequences in species that are known to hybridize, such as the white-headed gulls (Crochet et al. 2003) and Mallard/Black Ducks (Ankney et al. 1986; Avise et al. 1990).

In other cases, a lack of COI divergence may indicate that populations are part of a single species, helping to sort out misleading morphological classifications. For example, the blue and white morphs of *Chen caerulescens,* Snow Goose, were thought to be different species until recently (Cooke et al. 1995). The close COI similarity of American and Black Oystercatchers revealed in this study is consistent with suggestions that these are allopatrically distributed color morphs of a single species (Jehl 1985). Low COI divergences between American and Northwestern Crows similarly support earlier suggestions that these taxa are conspecific (Sibley and Monroe 1990; Madge and Burn 1994).

Just as COI similarities among species already questioned by taxonomists may reinforce these queries, deep COI divergences within species may reinforce suspicions of hidden diversity. For example, three of the four polytypic species in this study (Eastern Meadowlark, Marsh Wren, and Warbling Vireo) are split into two by some taxonomists (Wells 1998), and the fourth, Solitary Sandpiper, contains two allopatric subspecies with morphological differences (Godfrey 1976). In these cases, suspicions in the minds of taxonomists are reinforced by large COI divergences. If these species had not been the subject of prior scrutiny, COI barcoding would have flagged them as deserving of such attention.

The importance of sampling multiple individuals within each species is highlighted by a recent review which found evidence of species-level paraphyly or polyphyly in 23% of 2,319 animal species, including 16.7% of 331 bird species (Funk and Omland 2003). This review provides a clear discussion of possible causes (imperfect taxonomy, hybridization, incomplete lineage sorting) and indicates the need for the careful reexamination of current taxonomy and for the collection of genetic data across both geographic ranges and morphological variants. Barcoding, together with related developments in sequencing technology, is likely to provide an efficient approach to the assembly of such genetic data.

We expect that the assembly of a comprehensive barcode library will help to initiate taxonomic investigations that will ultimately lead to the recognition of many new avian species. This process will begin with the discovery of novel COI barcodes. Some of these cases will simply represent the first barcode records for described but previously unanalyzed species, but taxonomic study will confirm that others derive from new species. We propose that specimens with barcodes diverging deeply from known taxa should be known by a “provisional species” designation that links them to the nearest established taxon. For example, the divergent clusters of Solitary Sandpiper specimens might be called T. solitaria PS-1 and T. solitaria PS-2, highlighting a need for further taxonomic study.

What threshold might be appropriate for flagging genetically divergent specimens as provisional species? This threshold should certainly be high enough to separate only specimens that very likely belong to different species. Because patterns of intraspecific and interspecific variation in COI appear similar in various animal groups (Grant and Bowen 1998 [sardines]; Hebert et al. 2003a [moths]; Hogg and Hebert 2004 [springtails]), we propose a standard sequence threshold: 10× the mean intraspecific variation for the group under study. If applied to the birds examined in this study (0.27% average intraspecific variation; 2.7% threshold), a 10× threshold would recognize over 90% of the 260 known species, as well as the four probable new species. As this result demonstrates, a threshold approach will overlook species with short evolutionary histories and those exposed to recent hybridization, but it will be a useful screening tool, especially for groups that have not received intensive taxonomic analysis.

For 260 of the 667 bird species breeding in North America, our evidence shows that COI barcodes separate individuals into the categories that taxonomists call species. This adds to the evidence already in hand for insects and other arthropods that barcodes can be an efficient tool for species identification. Should future studies broaden this evidence, a comprehensive library of barcodes will make it easier to probe varied areas of avian biology. A DNA barcode will help, for example, when morphological diagnoses are difficult, as when identifying remnants (including eggs, nestlings, and adults) in the stomachs of predators. A DNA barcode could similarly identify fragments of birds that strike aircraft (Dove 2000) and recognize carcasses of protected or regulated species (Guglich et al. 1994). DNA barcodes could also reveal the species of avian blood in mosquitoes carrying West Nile virus (Michael et al. 2001; Lee et al. 2002), help experts distinguish morphologically similar juveniles or nonbreeding adults in banding work, and allow expanded nonlethal study of endangered or threatened populations.

The two essential components for an effective DNA barcode system (and thus a new master key to the encyclopedia of life [Wilson 2003]) are standardization on a uniform barcode sequence, such as COI, and a library of sequences linked to named voucher specimens. The present study provides an initial set of COI barcodes for about 40% of North American birds. More detailed sampling of COI sequences is needed for these species, and barcodes need to be gathered for the remaining North American birds and for those in other geographic regions. This work could represent a first step toward a DNA barcode system for all animal and plant life, an initiative with potentially widespread scientific and practical benefits (Stoeckle 2003; Wilson 2003; Blaxter 2004; Janzen 2004).

## Materials and Methods

### 

#### 

Existing data can only yield limited new insights into the effectiveness of a DNA-based identification system for birds. Two mitochondrial genes, cyt *b* and COI, are rivals for the largest number of animal sequence records greater than 600 bp in GenBank (4,791 and 3,009 species, respectively). However, COI coverage for birds is modest; 173 species share COI sequences with 600-bp overlap. As these records derive from a global avifauna of 10,000 species, they provide a limited basis to evaluate the utility of a COI-based identification system for any continental fauna, impelling us to gather new sequences.

We employed a stratified sampling design to gain an overview of the patterns of COI sequence divergence among North American birds. The initial level of sampling examined a single individual from each of 260 species to ascertain COI divergences among species. These species were selected on the basis of accessibility without regard to known taxonomic issues. The second level of sampling examined one to three additional individuals from 130 of these species to provide a general sense of intraspecific sequence divergences, as well as a preliminary indication of variation in each species. When possible, these individuals were obtained from widely separated localities in North America. The third level of our analysis involved sequencing four to eight more individuals for the few species where the second level detected more than 2% sequence divergence among individuals. Our studies examined specimens collected over the last 20 years; 98% were obtained from the tissue bank at the Royal Ontario Museum, Toronto, Canada. Collection localities and other specimen information are available in the “Birds of North America” file in the Completed Projects section of the Barcode of Life website (http://www.barcodinglife.com). Taxonomic assignments follow the latest North American checklist (AOU 1998) and its recent supplements (Banks et al. 2000, 2002, 2003).

Mitochondrial pseudogenes can complicate PCR-based studies of mitochondrial gene diversity (Bensasson et al. 2001; Thalmann et al. 2004). We used protocols to reduce pseudogene impacts that included extracting DNA from tissues rich in mitochondria (Sorenson and Quinn 1998), employing primers with high universality (Sorenson and Quinn 1998), and amplifying a relatively long PCR product because most pseudogenes are short (Pereira and Baker 2004). DNA extracts were prepared from small samples of muscle using the GeneElute DNA miniprep Kit (Sigma, St. Louis, Missouri, United States), following the manufacturer's protocols. DNA extracts were resuspended in 10 μl of H_2_O, and a 749-bp region near the 5′ terminus of the COI gene was amplified using primers (BirdF1-TTCTCCAACCACAAAGACATTGGCAC and BirdR1-ACGTGGGAGATAATTCCAAATCCTG). In cases where this primer pair failed, an alternate reverse primer (BirdR2-ACTACATGTGAGATGATTCCGAATCCAG) was generally combined with BirdF1 to generate a 751-bp product, but a third reverse primer (BirdR3-AGGAGTTTGCTAGTACGATGCC) was used for two species of *Falco.* The 50-μl PCR reaction mixes included 40 μl of ultrapure water, 1.0 U of *Taq* polymerase, 2.5 μl of MgCl_2_, 4.5 μl of 10× PCR buffer, 0.5 μl of each primer (0.1 mM), 0.25 μl of each dNTP (0.05 mM), and 0.5–3.0 μl of DNA. The amplification regime consisted of 1 min at 94 °C followed by 5 cycles of 1 min at 94 °C, 1.5 min at 45 °C, and 1.5 min at 72 °C, followed in turn by 30 cycles of 1 min at 4 °C, 1.5 min at 51 °C, and 1.5 min at 72 °C, and a final 5 min at 72 °C. PCR products were visualized in a 1.2% agarose gel. All PCR reactions that generated a single, circa 750-bp, product were then cycle sequenced, while gel purification was used to recover the target gene product in cases where more than one band was present. Sequencing reactions, carried out using Big Dye v3.1 and the BirdF1 primer, were analyzed on an ABI 377 sequencer. The electropherogram and sequence for each specimen are in the “Birds of North America” file, but all sequences have also been deposited in GenBank (see Supporting Information). COI sequences were recovered from all 260 bird species and did not contain insertions, deletions, nonsense, or stop codons, supporting the absence of nuclear pseudogene amplification (Pereira and Baker 2004). In addition to 429 newly collected sequences, nine GenBank sequences from five species were included (these were the only full-length COI sequences corresponding to species in this study).

Sequence divergences were calculated using the K2P distance model (Kimura 1980). A NJ tree of K2P distances was created to provide a graphic representation of the patterning of divergences among species (Saitou and Nei 1987).

## Supporting Information

Figure S1 Birds Appendix Complete NJ tree based on K2P distances at COI for 437 sequences from 260 species of North American birds. Entries marked with an asterisk represent COI sequences from GenBank.
(100 KB PDF).

### Accession Numbers

Sequences described in Materials and Methods have been deposited in GenBank under accession numbers AY666171 to AY666596.

## Abbreviations

COI: cytochrome *c* oxidase I
K2P: Kimura-2 parameter
mtDNA: mitochondrial DNA
NJ: neighbor joining
