# Supplementary figures and images for: Identification of Birds through DNA Barcodes
Source: PLoS Biol. 2004 Sep 28;2(10):e312. doi: 10.1371/journal.pbio.0020312 (PMC518999; doi:10.1371/journal.pbio.0020312)

2%

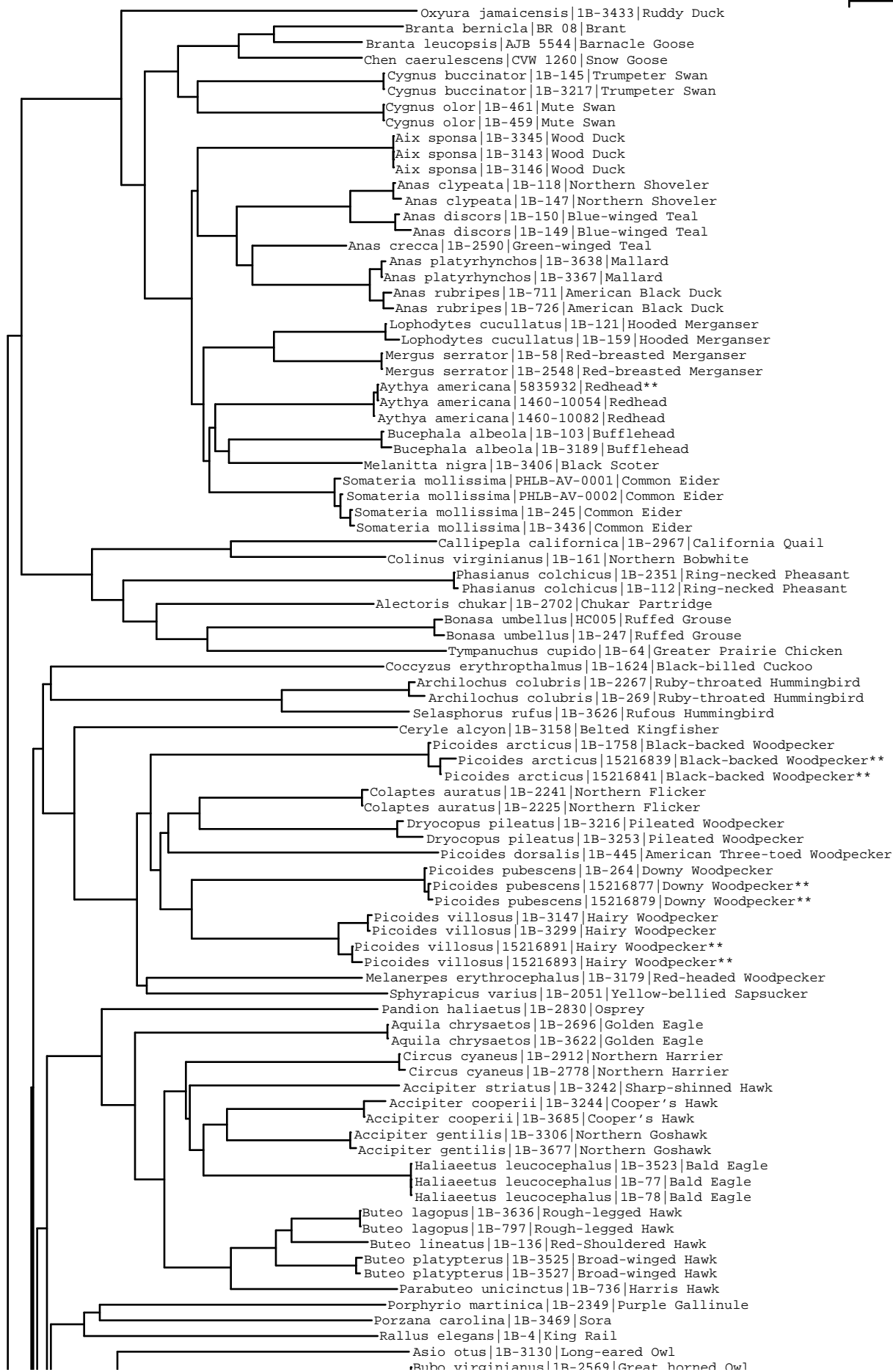

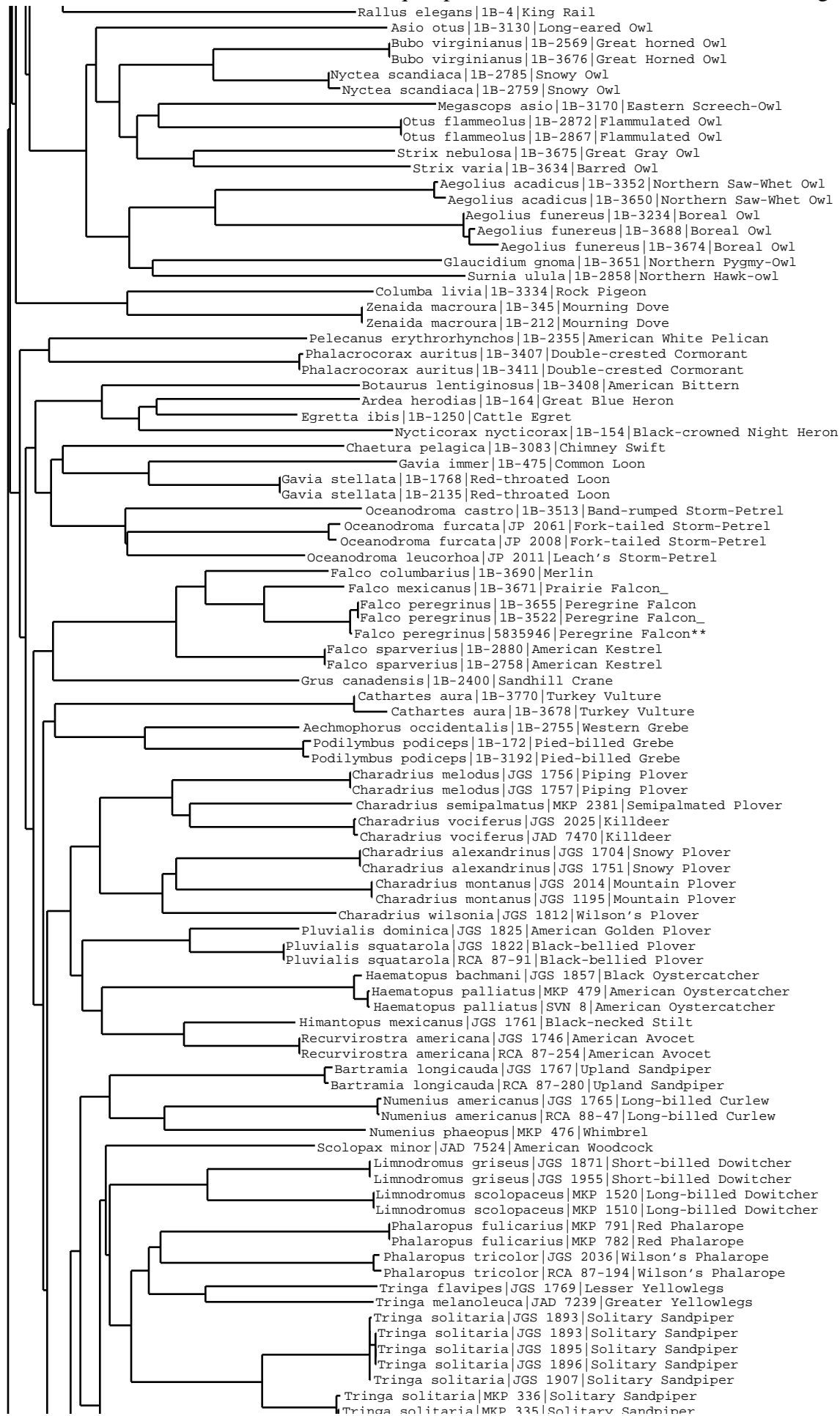

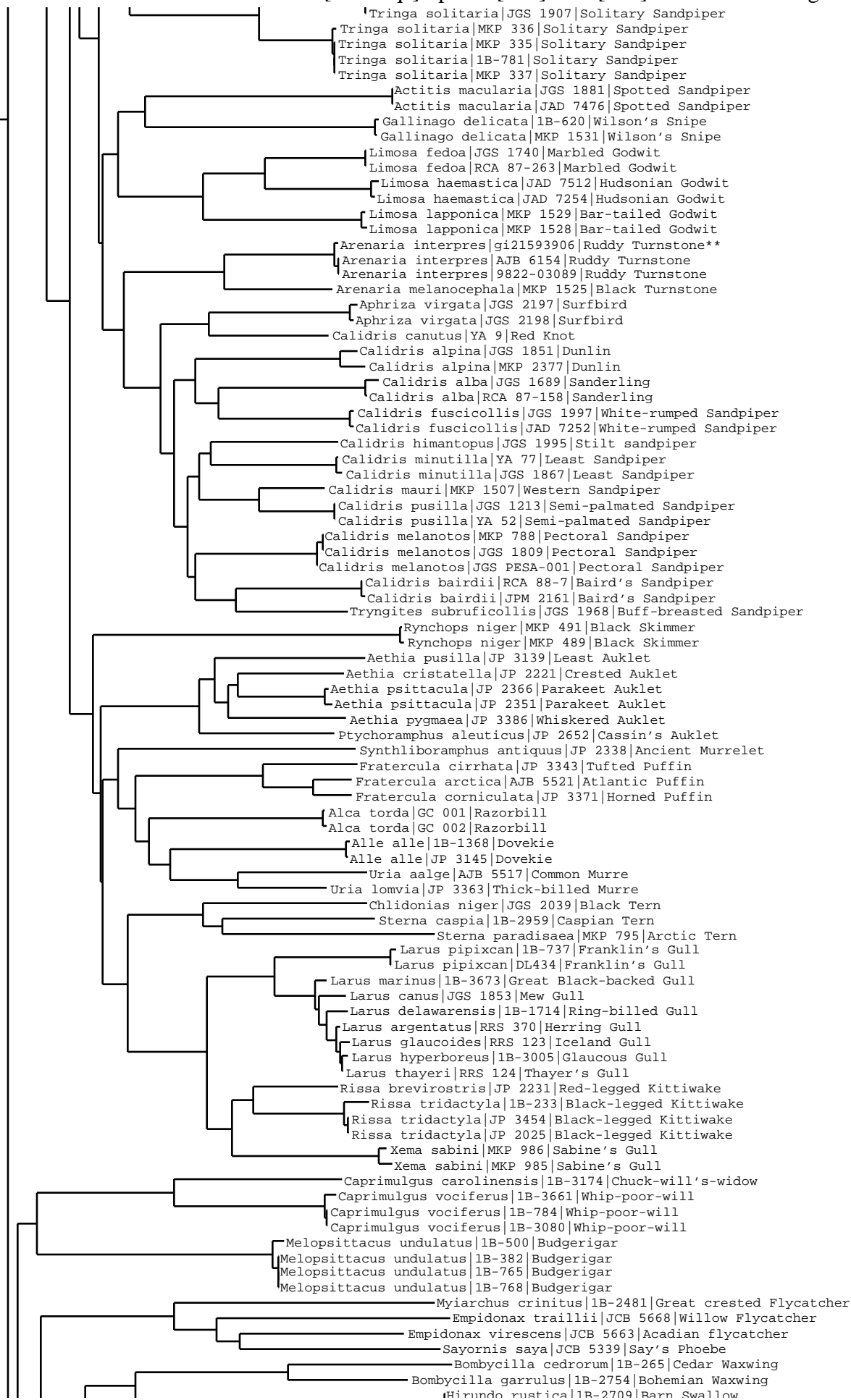

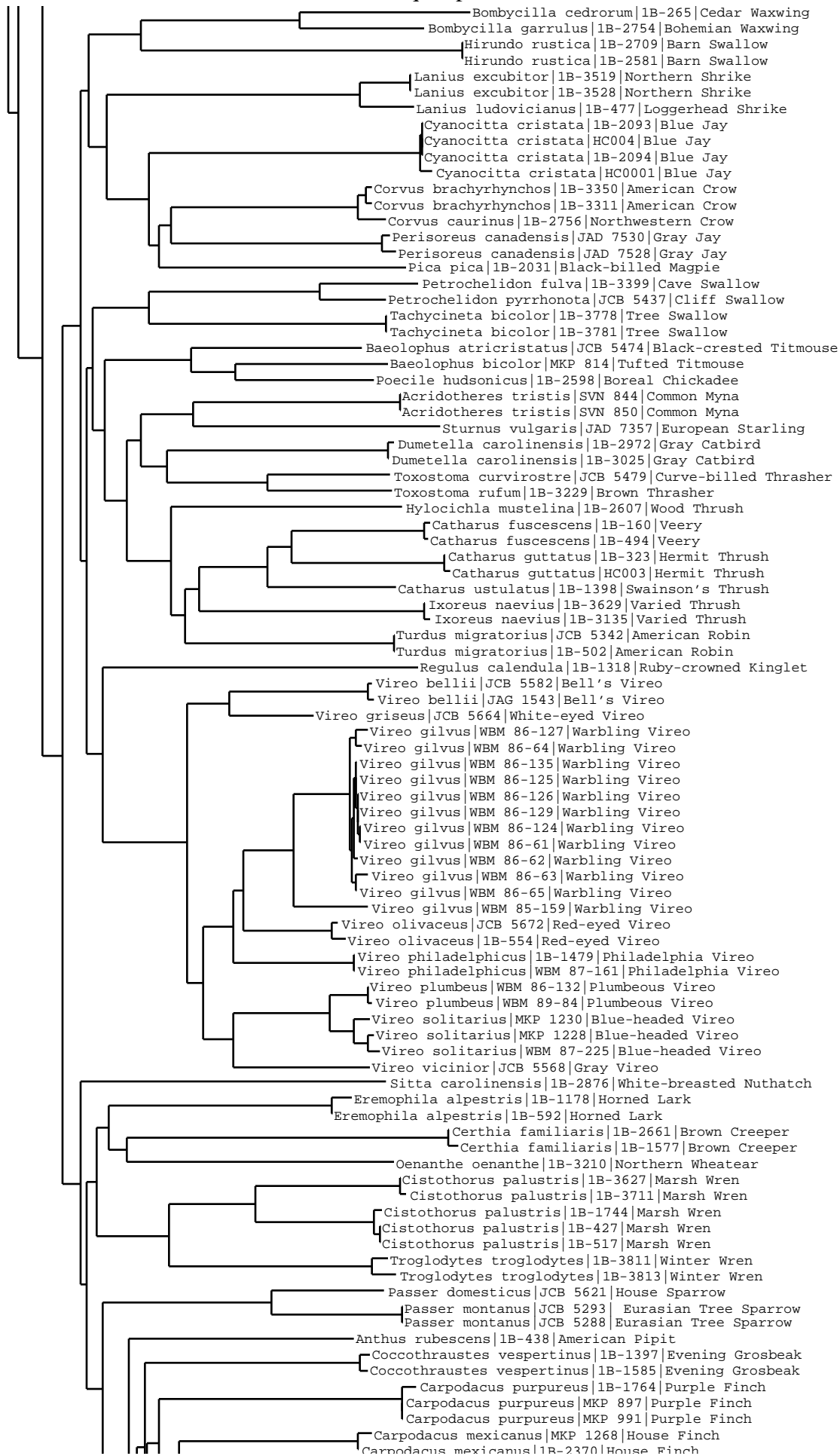

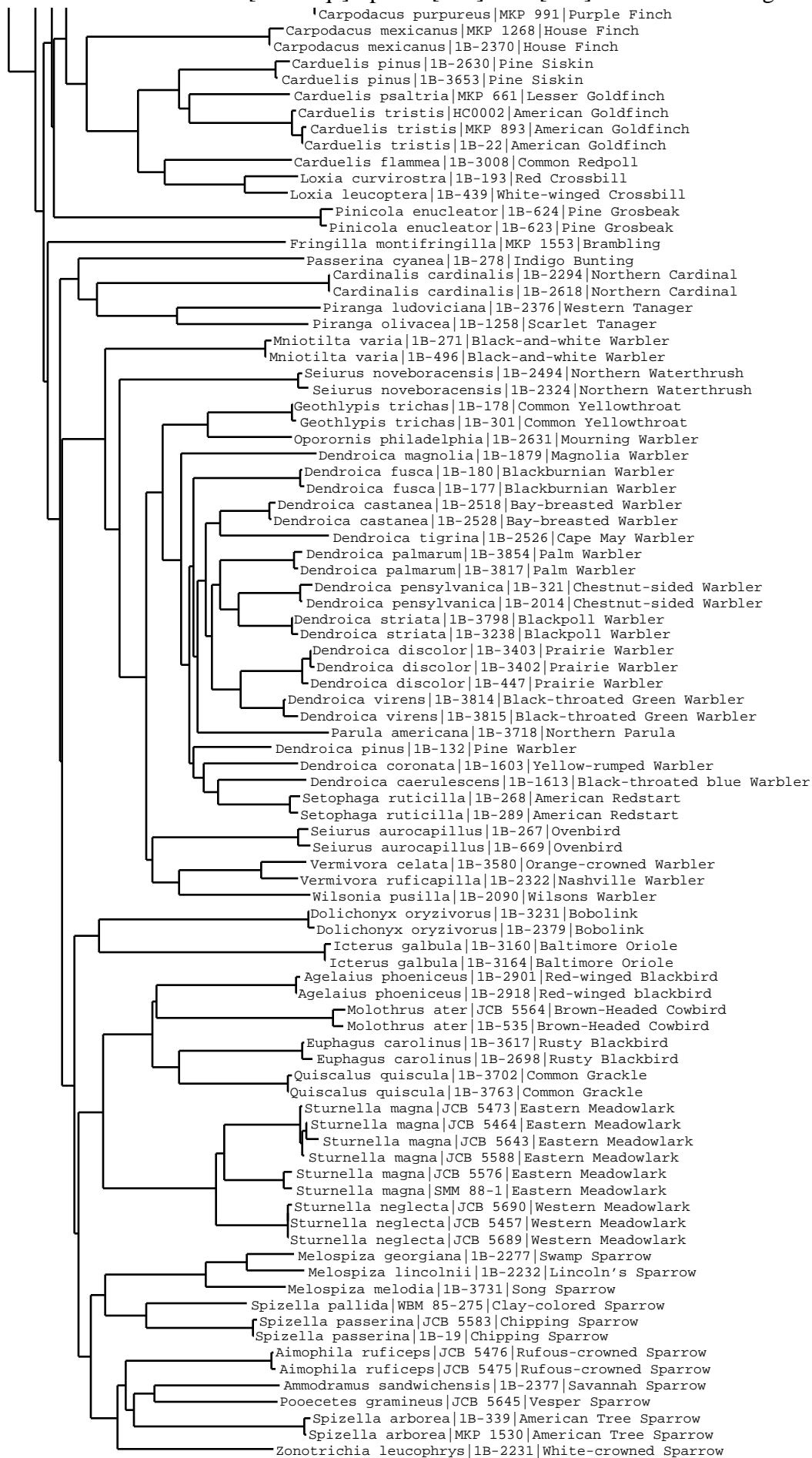

Supplement: Figure S1 — Complete NJ tree based on K2P distances at COI for 437 sequences from 260 species of North American birds. Entries marked with an asterisk represent COI sequences from GenBank. (100 KB PDF). [file pbio.0020312.sg001.pdf]
